# Vortioxetine Differentially Modulates MK-801-Induced Changes in Visual Signal Detection Task Performance and Locomotor Activity

**DOI:** 10.3389/fphar.2018.01024

**Published:** 2018-09-13

**Authors:** Todd M. Hillhouse, Christina R. Merritt, Douglas A. Smith, Manuel Cajina, Connie Sanchez, Joseph H. Porter, Alan L. Pehrson

**Affiliations:** ^1^Department of Psychology, Weber State University, Ogden, UT, United States; ^2^Department of Psychology, Virginia Commonwealth University, Richmond, VA, United States; ^3^Lundbeck Research USA, Inc., Paramus, NJ, United States; ^4^Department of Clinical Medicine, Aarhus University, Aarhus, Denmark; ^5^Department of Psychology, Montclair State University, Montclair, NJ, United States

**Keywords:** vortioxetine, MK-801, visual signal detection task, locomotor activity, attention

## Abstract

Attention impairment is a common feature of Major Depressive Disorder (MDD), and MDD-associated cognitive dysfunction may play an important role in determining functional status among this patient population. Vortioxetine is a multimodal antidepressant that may improve some aspects of cognitive function in MDD patients, and may indirectly increase glutamate neurotransmission in brain regions classically associated with attention function. Previous non-clinical research suggests that vortioxetine has limited effects on attention. This laboratory previously found that vortioxetine did not improve attention function in animals impaired by acute scopolamine administration, using the visual signal detection task (VSDT). However, vortioxetine has limited effects on acetylcholinergic neurotransmission, and thus it is possible that attention impaired by other mechanisms would be attenuated by vortioxetine. This study sought to investigate whether acute vortioxetine administration can attenuate VSDT impairments and hyperlocomotion induced by the non-competitive *N*-methyl-D-aspartate (NMDA) receptor antagonist MK-801. We found that acute vortioxetine administration had no effect on VSDT performance on its own, but potentiated MK-801-induced VSDT impairments. Furthermore, vortioxetine had no effect on locomotor activity on its own, and did not alter MK-801-induced hyperlocomotion. We further investigated whether vortioxetine’s effect on MK-801 could be driven by a kinetic interaction, but found that plasma and brain exposure for vortioxetine and MK-801 were similar whether administered alone or in combination. Thus, it appears that vortioxetine selectively potentiates MK-801-induced impairments in attention without altering its effects on locomotion, and further that this interaction must be pharmacodynamic in nature. A theoretical mechanism for this interaction is discussed.

## Introduction

Major Depressive Disorder (MDD) is a highly prevalent psychiatric illness that is associated with marked functional impairment (McCall and Dunn, 2003), and is a leading cause of disability worldwide (Collins et al., 2011). Although MDD is primarily seen as a disorder of mood, extant clinical data suggests that MDD patients commonly present with severe impairments in cognitive processes such as executive function and attention (McIntyre et al., 2013). One study estimated that more than a third of MDD patients are functioning at least two standard deviations below average in one or more cognitive domains (Gualtieri and Morgan, 2008), and several research groups have found predictive relationships between MDD-related cognitive dysfunction and either functional disability or poor perceived workplace performance (McCall and Dunn, 2003; Jaeger et al., 2006; McIntyre et al., 2013). Although many believe that these cognitive impairments withdraw when mood symptoms are effectively treated, there is some evidence that counters this narrative. For example, Hasselbalch et al. (2011) conducted a systematic review of studies that investigated cognitive function in remitted MDD patients, and reported that significant impairment in tests of one or more cognitive domains (attention, executive function, memory, or global function) was observed in 9 of the 11 studies. These observations suggest that treatment of MDD-related mood dysfunction alone is insufficient to engender full functional recovery for many MDD patients. Therefore, identifying biological mechanisms capable of remediating MDD-associated impairments in cognitive function is an important strategic goal to improve functional outcomes in MDD patients.

Vortioxetine is an antidepressant featuring a complex pharmacological profile consisting of inhibition at the serotonin (5-HT) transporter (SERT), antagonism at 5-HT_1D_, 5-HT_3_, and 5-HT_7_ receptors, partial agonism at 5-HT_1B_ receptors, and agonism at 5-HT_1A_ receptors. Clinical evidence from several randomized placebo-controlled trials suggests that vortioxetine attenuates impairments in executive function and speed of processing (McIntyre et al., 2016). Additionally, in a predefined secondary outcome in one randomized, placebo-controlled trial, vortioxetine significantly improved scores in the perceived deficits questionnaire attention/concentration subscale (Mahableshwarkar et al., 2015), a subjective measure of attention performance. Non-clinical studies have also demonstrated that vortioxetine improves cognitive performance in a variety of rodent-based models of executive function. Wallace et al. (2014) demonstrated that vortioxetine attenuated impairments in the attentional set shifting task (AST) induced by 5-HT depletion or chronic intermittent cold stress in rats. Additionally, this laboratory recently demonstrated that vortioxetine reverses AST impairments induced by subchronic administration of the non-competitive *N*-methyl-D-aspartate (NMDA) receptor antagonist phencyclidine (Pehrson et al., 2018).

Although non-clinical literature supports the idea that vortioxetine improves behavioral measures of executive function in deficit models, the non-clinical data on vortioxetine’s influence on attention has been equivocal at best. A recent study investigated vortioxetine’s effects on event-related potentials (ERPs) assessed by electroencephalographic recordings during a two-tone auditory discrimination task (Laursen et al., 2017). Laursen et al. (2017) found that, while acute vortioxetine administration did not alter behavioral responses to target and oddball tones, it did significantly increase the amplitude of hippocampal P3 ERPs, an analog of the human P300 ERP that is putatively relevant for attention (Gray et al., 2004). Thus, vortioxetine may increase some aspects of hippocampal synchrony during attention-relevant tasks.

However, a recent paper from this laboratory investigated the effects of vortioxetine in another model of attention, visual signal detection task (VSDT) impairments induced by the muscarinic acetylcholinergic receptor antagonist scopolamine. This study demonstrated that, although acute vortioxetine treatment was able to reverse scopolamine-induced deficits in memory performance at clinically relevant doses, it was unable to alter scopolamine-induced impairments in the VSDT (Pehrson et al., 2016a). Importantly, acute administration of the acetylcholinesterase inhibitor donepezil was able to significantly attenuate scopolamine-induced VSDT impairments in this study (Pehrson et al., 2016a). As an assessment of vortioxetine’s general effects on attention function, this study could be questioned based on the use of scopolamine as a platform for inducing attention impairment. Although previous research has consistently demonstrated that acute vortioxetine treatment can modulate cholinergic neurotransmission by increasing acetylcholine efflux (Mork et al., 2013; Pehrson et al., 2016a), this increase is also consistently small and short-lived. Thus, vortioxetine’s lack of an effect on scopolamine-induced VSDT impairments may be related to its limited influence on cholinergic neurotransmission.

One of several theories on the mechanism by which vortioxetine influences cognitive function suggests that vortioxetine administration indirectly increases glutamate neurotransmission in brain regions such as the medial prefrontal cortex (Riga et al., 2016, 2017), a brain region associated with attention function in tasks such as the 3 choice serial reaction time task (Totah et al., 2009). While this theory has accumulated some supporting mechanistic evidence, there are still relatively few studies that have directly evaluated a role for altered glutamatergic neurotransmission in vortioxetine’s effects on cognitive function. Thus, the current work was a part of a multi-study project aimed at evaluating vortioxetine’s effects on frontal cortex-dependent cognitive impairments induced by dysregulation of glutamate neurotransmission. Elsewhere, we have reported a study evaluating vortioxetine’s effects on subchronic PCP-induced impairments in the AST, a test of executive function (Pehrson et al., 2018). Given that we are unaware of any studies that have evaluated a role for this mechanism in behavioral measures of attention, the goal of the present study was to evaluate vortioxetine’s effects on VSDT impairments induced by acute administration of a non-competitive NMDA receptor antagonist. Therefore, we decided to investigate vortioxetine’s effects on VSDT deficits induced by acute MK-801 administration, which has well-characterized effects in this task (see Discussion, below). In addition, given that the pattern of 5-HT_3_ receptor expression is relatively circumscribed in the forebrain, and is not strongly present in motor function circuits such as the striatum (Raffa et al., 1989; Morales et al., 1998; Pehrson et al., 2016b), vortioxetine’s effects on glutamate neurotransmission should be similarly limited to frontal cortex-dependent tasks, and would not be present in assessments of motor function. Therefore, we made the following hypotheses: (1) vortioxetine will reverse behavioral impairments in the VSDT (a test of attention) induced by the NMDA receptor antagonist MK-801, and (2) vortioxetine will not alter locomotor activity induced by MK-801.

## Materials and Methods

### Subjects

A total of 55 adult male Sprague-Dawley rats (Harlan Laboratories Inc., Frederick, MD, United States) weighing between 225 and 350 g at the start of the experiment. Of these, 15 rats were used in the VSDT experiment (Harlan Laboratories Inc., Frederick, MD, United States), 16 rats were used for the locomotor activity experiment (Harlan Laboratories), and 24 rats were used for drug exposure studies (Charles River Laboratories, Wilmington, MA, United States). Rats were housed individually in a temperature- and humidity-controlled environment on a 12-h/12-h light/dark cycle (lights on 0600 h). All experimental procedures were conducted during the light portion of the cycle. Except where noted below, animals were given *ad libitum* access to food and water. In addition, upon arrival at the vivarium, all rats were given a 5–7-day acclimation period before any experimental procedures began. All experimental procedures were approved by the Institutional Animal Care and Use Committee at Virginia Commonwealth University or Lundbeck Research USA, Inc. prior to the start of experiments, and were conducted in accordance with National Institutes of Health *Guide for the Care and Use of Laboratory Animals* (Institute of Laboratory Animal Resources, 2011).

### Drugs and Chemicals

Vortioxetine HBr was obtained from Lundbeck Research USA, Inc. (+) MK-801 hydrogen maleate was obtained from Sigma Aldrich (St. Louis, MO, United States), while 2-hydroxypropyl-beta-cyclodextrin was obtained from Roquette America (Keouk, IA, United States). Vortioxetine was dissolved in a 20% (w/v) solution of 2-hydroxypropyl-beta-cyclodextrin (20%CD) at 5 mg/mL concentration and injected subcutaneously (s.c.) at a 2 mL/kg volume, for a final dose of 10 mg/kg. MK-801 was dissolved in water at 0.1 or 0.2 mg/mL and injected s.c at a 1 mL/kg volume, for a final dose of 0.1 or 0.2 mg/kg. The specific doses and injection timing for each experiment are discussed below. All doses refer to the mass of the free base, not the salt.

### Apparatus

#### Visual Signal Detection Task

Training and experimental sessions were conducted in four standard operant chambers enclosed within sound attenuating cubicles (Med-Associates). Each chamber was equipped with a signal light, food hopper, two retractable levels, and a house light. The front panel of the operant chamber housed the signal light which was mounted directly above a food hopper and two retractable levers positioned on either side of the food cup. Additionally, the back panel was equipped with a house light that provided a background illumination (except during a time out period).

#### Locomotor Activity

Habituation and experimental sessions were conducted in four standard open field activity chambers enclosed within sound attenuating cubicles (Med-Associates). Each chamber was equipped with three 16-beam IR arrays, which tracked the rats throughout the chambers, and a house light. Activity Monitor (version 7; Med-Associates) was used to collect the data provided from the three 16-beam IR arrays.

#### Drug Exposure Studies

Drug exposure in plasma and brain tissue was measured using an Aria TLX2 liquid chromatography system (Thermo Electron, San Jose, CA, United States) coupled with a TSQ Quantum Ultra mass spectrometer (Thermo Electron).

### Behavioral Procedures

#### Visual Signal Detection Task

##### Food restriction

Prior to the start of VSDT experiments, rats were maintained on free-feeding as described above. Based on weights measured during this period, a target weight was calculated for each individual, set to 85% of free-feeding weight. Daily access to food was restricted so each rat’s actual body weight closely approximated the calculated target weight. Water was available *ad libitum* in home cages at all times.

##### VSDT training

The rats were trained and tested according to procedures consistent with previously published experiments (Hillhouse and Prus, 2013; Hillhouse et al., 2015; Pehrson et al., 2016a). Each trial started with both the house light and a variable-intensity signal light on. The combined background illumination of these lights during this phase of training was 0.9 lux. During training, each trial started with a consistent pre-signal interval of 4 s, during which the lighting intensity was unchanged. After the pre-signal interval, rats experienced either a “blank” or “signal” trial for 500 ms. For blank trial conditions, there was no change in the signal light intensity for 500 ms. However, signal trial conditions featured a 1.5 lux increase in illumination intensity of the signal light for 500 ms (to a total intensity of 2.4 lux). A post-signal interval of 1 s followed the signal (or blank) segment of the trial during which time the chamber remained at the 0.9 lux background illumination.

Following the post-signal interval, the left and right levers were extended into the chamber. If a rat pressed the signal-lever (randomly assigned as either the right or left lever) after a signal trial it was recorded as a “hit” and the rat received a food pellet. If a rat pressed the blank-lever during a blank trial it was recorded as a correct rejection and the rat received a food pellet. Levers were retracted after a response or 5 s (whichever occurred first). If rats failed to make a response in 5 s, then it was counted as an omission. Incorrect responses (i.e., blank-lever during a signal trial or signal-lever during a blank trial) and trial omissions resulted in no food pellet delivery and a 2 s time-out (all lights in test chamber turned off including the house light). Rats were trained until a criterion of ≥70% choice accuracy was obtained for 3 consecutive sessions.

##### VSDT testing

Test sessions were identical to training sessions except that 3 signal intensities were used (i.e., 0.4, 0.6, and 1.5 lux increase above blank conditions, order randomized) and pre-signal interval delays of 3, 6, and 12 s (order randomized) were used. Test sessions consisted of 90 blank trials and 90 signal trials (i.e., 30 low, 30 moderate, and 30 high signal intensity trials). Animals received at least one training session immediately preceding a test session. Test sessions were conducted no more than twice a week (typically Tuesdays and Fridays) and were separated by at least 72 h to allow for drug washout. On test days, animals were injected with vehicle [20% (w/v) aqueous solution of parenteral grade 2-hydroxypropyl-beta-cyclodextrin, 60 min s.c.] or vortioxetine (10 mg/kg, 60 min s.c.) followed by either MK-801 (0.1 mg/kg, 30 min s.c.) or vehicle (0.9% saline 30 min s.c.) prior to test session start. The order of drug combinations was determined by a randomized Latin-square design, and thus each animal in the VSDT study was present in each treatment condition. Drug doses and pretreatment times were based on published literature. In the case of MK-801, the doses and injection timing have previously demonstrated robust behavioral effects in the VSDT without producing pronounced motor suppression (Rezvani et al., 2008; Levin et al., 2011; Hillhouse and Porter, 2014; Leiser et al., 2014; Hillhouse et al., 2015; Pehrson et al., 2016a). Preliminary experiments demonstrated that lower doses of MK-801failed to produce reliable behavioral impairments. Thus, these lower doses were deemed to be unacceptable for use in a randomized Latin square experimental design. In the case of vortioxetine, the 10 mg/kg (1 h s.c.) dose and injection timing was chosen because it has been extensively characterized in pharmacological, neurochemical, and behavioral cognition models, and is thought to represent the top of the clinically relevant dose range from a mechanistic perspective (Pehrson et al., 2013b, 2016a, 2018; du Jardin et al., 2014; Jensen et al., 2014; Leiser et al., 2014).

#### Locomotor Activity (LMA) Studies

##### LMA Training

Each rat received a total of seven habituation training sessions over the course of 2 weeks. For these habituation sessions, rats were placed in the center of the open field chamber and were allowed to explore for 60 min. After the seven habituation sessions, each rat was moved to the experimental phase of the study.

##### LMA Testing

Test sessions consisted of three separate phases. The first phase consisted of a 60-min habituation period in which animals were placed in activity chambers and were monitored without the influence of a drug. After the habituation period, rats received an injection of either 20%CD (2 mL/kg, s.c.) or 10 mg/kg vortioxetine (s.c.) and immediately placed back into the open field chamber for the second activity monitoring phase, which lasted 30 min. Immediately after the second monitoring phase was completed, rats were given a second injection that consisted of either saline (1 mL/kg, s.c.) or 0.2 mg/kg MK-801 (s.c.). The 0.2 mg/kg MK-801 dose was chosen on the basis of evidence that it induces robust hyperlocomotion (Ogren and Goldstein, 1994; Scorza et al., 2008), while lower doses produce less reliable effects on locomotion (Amalric et al., 1994; Druhan et al., 1996; Scorza et al., 2008). Rats were immediately placed back into the activity chamber for 90 min. Locomotor testing was conducted using a within-subjects design, with at least a 1-week washout period between sessions. The order of drug combinations was determined by a randomized Latin-square design, and thus each animal in the LMA study was present in each treatment condition locomotor chambers were cleaned between each group of animals.

### Drug Exposure Study Methods

In order to investigate whether vortioxetine and MK-801 have any pharmacokinetic interactions, studies were conducted to examine vortioxetine and MK-801 exposure in plasma and brain tissue. Rats in this experiment were randomly assigned to one of three groups. In the first group, rats received an injection of 20%CD (2 mL/kg, 60 min s.c.) followed by 0.1 mg/kg MK-801 (30 min s.c.). Rats in the second group received injections of 10 mg/kg vortioxetine (60 min s.c.) followed by saline (1 mL/kg, 30 min s.c.). The final group received injections of 10 mg/kg vortioxetine (60 min s.c.) followed by 0.1 mg/kg MK-801 (30 min s.c.). These injection times are identical to those used in behavioral testing.

At the appropriate time after injection, rats were deeply anesthetized using CO_2_ and killed by decapitation. Blood and brain tissue were collected and processed as described elsewhere (Pehrson et al., 2016a). Vortioxetine and MK-801 exposure levels were detected using a liquid chromatography/mass spectrometry system. The methods used for detecting exposure levels were as described previously (Pehrson et al., 2016a), with one modification. Spectra for MK-801 were acquired in positive selected reaction monitoring mode with the following settings: the parent mass (222.13 m/z) and daughter ion 1 (203 m/z) were detected at 40 collision energy (CE) with a tube lens of 115. Daughter ion 2 (205 m/z) was detected at 25 CE with tube lens set at 115.

### Statistics

#### Visual Signal Detection Task

The dependent variables for the VSDT task were as follows: (1) Percent hits = (number of correct responses on signal trials/the number of signal trials completed) × 100. (2) Percent correct rejections = (number of correct responses on blank trials/the number of blank trials completed) × 100. (3) Response latency = total time elapsed from when the levers were extended to when a lever press occurred/the number of trials completed (these data were collapsed for signal and blank trials). (4) Response omissions = total number of trials where no response occurred (these data were collapsed across signal and blank trials). A two-factor repeated measures ANOVA was conducted for percent hit with signal intensity and treatment condition as factors. One-way repeated measures ANOVAs were used to assess the effect of treatment condition on percent correct rejections, response latency, or trial omissions.

#### Locomotor Activity

The dependent variables for the locomotor activity studies were as follows: horizontal beam breaks and rearing (vertical beam breaks). These dependent measures were analyzed using two-factor repeated measures ANOVAs, with behavioral epoch and treatment as within-subjects factors. Where appropriate, these ANOVAs were followed by Tukey *post hoc* tests.

#### Drug Exposure

MK-801 and vortioxetine concentrations in plasma were expressed in nM or μM, respectively, while concentrations in brain tissue were expressed in nmol/kg or μmol/kg. Brain to plasma exposure ratios were calculated by dividing a given brain concentration by the relevant plasma concentration. Statistical analysis of exposure data was conducted using independent samples *t*-tests.

## Results

### Visual Signal Detection Task

**FIGURE 1:**
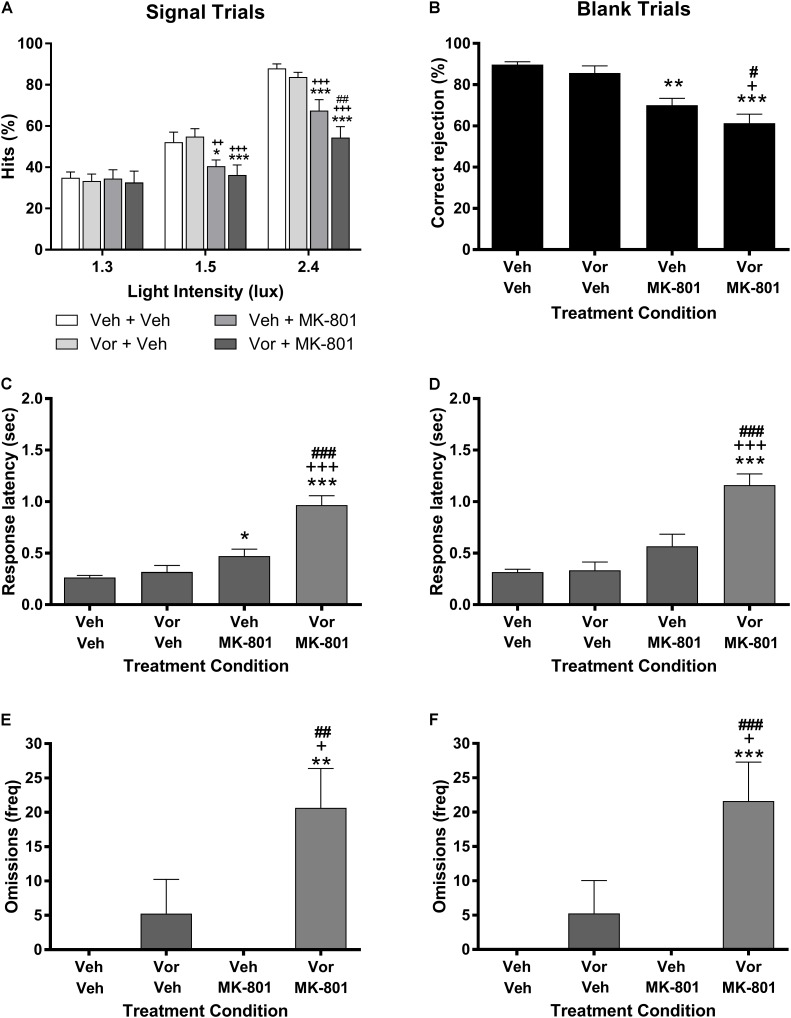
Vortioxetine potentiates MK-801-induced visual signal detection task impairments, but has no effects on its own. Left-side panels represent dependent measures related to signal trials, while those on the right represent dependent measures related to blank trials. **(A,B)** Represent hits or correct rejections, respectively. **(C,D)** Represent response latencies recorded during the relevant trial type, while **(E,F)** represent the frequency of omissions recorded during each trial type. 0.1 mg/kg MK-801 administration (30 min s.c.) induced significant, signal intensity-dependent impairments in hits **(A)**, correct rejections **(B)**, and increased response latencies during signal trials **(C)** compared to vehicle controls. 10 mg/kg vortioxetine (1 h s.c.) did not significantly alter performance in any dependent measure on its own. The combination of vortioxetine + MK-801 induced significantly greater impairments in hits, correct rejections, response latencies **(C,D)**, and omissions **(E,F)**. Asterisks represent significant differences from the Veh + Veh group (^∗^*P* < 0.05, ^∗∗^*P* < 0.01, ^∗∗∗^*P* < 0.001). Plus signs represent significant differences from the Vor + Veh group (^+^*P* < 0.05, ^++^*P* < 0.01, ^+++^*P* < 0.001). Number signs represent significant differences from the Veh + MK-801 group (^#^*P* < 0.05, ^##^*P* < 0.01, ^###^*P* < 0.001).

**Figure 1** represents the effects of vortioxetine, MK-801, and combinations thereof on VSDT performance. Analysis of the data on the percent hits dependent measure (**Figure 1A**) found a significant main effect of stimulus intensity light [*F*(2,28) = 130.5, *P* < 0.001] and treatment group [*F*(3,42) = 8.34, *P* < 0.001], as well as a significant interaction between these factors [*F*(6,84) = 7.25, *P* < 0.001]. Under control conditions, the expected stimulus intensity-dependent increase in percent hits was observed. The 10 mg/kg vortioxetine (1 h s.c.) + vehicle condition had no significant effects on performance by comparison to the control condition. Treatment with vehicle + 0.1 mg/kg MK-801 (30 min s.c.) induced significant impairments on the percent hits dependent measure compared to vehicle control at the 1.5 and 2.4 lux stimulus intensities, but not at the 1.3 lux stimulus intensity. The 10 mg/kg vortioxetine + 0.1 mg/kg MK-801 treatment group was also significantly impaired compared to vehicle controls at the 1.5 and 2.4 lux stimulus intensities, however, at the 2.4 lux stimulus intensity this group was also significantly more impaired than the vehicle + 0.1 mg/kg MK-801 treatment group. Similar effects were found on the remaining dependent variables. MK-801 (0.1 mg/kg) alone significantly reduced correct rejection accuracy [*F*(3,42) = 14.79, *P* < 0.001: **Figure 1B**] and significantly increased response latency for percent hit [*F*(3,42) = 35.62, *P* < 0.001: **Figure 1C**]; whereas, the combination of 10 mg/kg vortioxetine + 0.1 mg/kg MK-801 produced a greater impairments than MK-801 alone [*F*(3,42) = 7.52, *P* < 0.001; **Figure 1E**). The response omissions were evenly distributed across light intensity for MK-801 alone (1.3 lux = 2.07 ± 1.86; 1.5 lux = 1.73 ± 1.74; 2.4 lux = 1.47 ± 1.40) and the combination of vortioxetine and MK-801 (1.3 lux = 7.40 ± 2.04; 1.5 lux = 7.13 ± 2.00; 2.4 lux = 6.13 ± 1.98). The combination of vortioxetine and MK-801 produced a significant increase in the response latency for correct rejections [*F*(3,42) = 30.82, *P* < 0.001: **Figure 1D**] and response omissions [*F*(3,42) = 8.52, *P* < 0.001: **Figure 1F**]. Treatment with vortioxetine alone did not alter response latency (percent hit or correct rejection), or response omissions.

### Locomotor Activity

**Table 1** represents the effects of vortioxetine, MK-801, or combinations thereof on locomotor activity. Analysis of the horizontal activity dependent measure using two factor repeated measures ANOVAs found significant main effects for treatment group [*F*(3,45) = 22.07, *p* < 0.001] and behavioral epoch [*F*(2,30) = 30.94, *p* < 0.001], as well as a significant interaction between these factors [*F*(6,90) = 22.34, *p* < 0.001]. *Post hoc* analysis revealed that, within the baseline behavioral epoch, there were no significant differences between the treatment groups in terms of horizontal activity. Similarly, during the second behavioral epoch, after injection with either vehicle or vortioxetine, no significant differences in horizontal activity were detected. However, in the final behavioral epoch, after injection with either vehicle or MK-801, some notable differences were observed between treatment groups. Specifically, both the vehicle + MK-801 and the vortioxetine + MK-801 treatment groups exhibited significantly more horizontal activity than the vehicle + vehicle or vortioxetine + vehicle treatment groups (*p* < 0.001). No differences were observed between the vehicle + MK-801 and vortioxetine + MK-801 treatment groups.

**Table 1:** Vortioxetine does not alter MK-801-induced hyperactivity.

Behavioral epoch	Treatment group			
	Veh + Veh	Veh + MK-801	Vor + Veh	Vor + MK-801
**Horizontal activity**				
Baseline	1120 ± 117	1096 ± 156	1218 ± 112	1110 ± 108
After Veh or Vor	478 ± 43	485 ± 47	610 ± 52	599 ± 66
After Veh or MK-801	610 ± 83	16637 ± 3470^∗∗∗,+++^	654 ± 96	17318 ± 2865^∗∗∗,+++^
**Rearing**				
Baseline	99 ± 11	88 ± 10	100 ± 9	93 ± 11
After Veh or Vor	39 ± 4	38 ± 5	49 ± 4	44 ± 5
After Veh or MK-801	50 ± 8	111 ± 25^∗∗∗,++^	61 ± 10	89 ± 24^∗^

*Locomotor activity was observed in three contiguous epochs on a given test day: a 60-min baseline, a 30-min session starting immediately after vehicle or 10 mg/kg vortioxetine (s.c.), and a 90-min session starting immediately after vehicle or 0.2 mg/kg MK-801 (s.c). MK-801 administration induced significant increases in horizontal activity that was not modified by pretreatment with vortioxetine. No treatment induced significant changes in rearing behavior. Data are presented as Mean ± SEM. ^∗^*P* < 0.05 compared to the Veh + Veh treatment groups. Asterisks represent significant differences from the Veh + Veh group (^∗∗^*P* < 0.01, ^∗∗∗^*P* < 0.001). Plus signs represent significant differences from the Vor + Veh group (^+^*P* < 0.05, ^++^*P* < 0.01, ^+++^*P* < 0.001).*

Analysis of the rearing dependent measure using a two factor repeated measures ANOVA revealed a significant main effect for behavioral epoch [*F*(2,30) = 14.97, *p* < 0.001], but not for treatment group [*F*(3,45) = 1.18, n.s.]. However, a significant interaction between the treatment group and behavioral epoch factors was observed [*F*(6,90) = 3.04, *p* < 0.01]. Again, *post hoc* analysis failed to reveal any significant differences in rearing behavior between the treatment groups during the baseline epoch, or during the second epoch (after vehicle or vortioxetine administration). However, during the final behavioral epoch (after vehicle or MK-801 administration), we observed significant increases in rearing behavior in the vehicle + MK-801 group compared to the vehicle + vehicle (*p* < 0.001) and vortioxetine + vehicle (*p* < 0.01) treatment groups. The vortioxetine + MK-801 group also exhibited significantly more rearing behavior than the vehicle + vehicle group (*p* < 0.05), but was not significantly different from either the vehicle + MK-801 group or the vortioxetine + vehicle group.

### Drug Exposure

#### Vortioxetine Exposure

Drug exposure data after treatment with 10 mg/kg vortioxetine (1 h s.c.), 0.1 mg/kg MK-801 (30 min s.c.), or the combination are presented in **Table 2**. Exposure to vortioxetine in plasma [*t*(14) = 0.045, n.s.] and brain [*t*(14) = 0.11, n.s.] were similar whether examined in animals from the vortioxetine + vehicle or vortioxetine + MK-801 treatment groups. Additionally, the brain:plasma ratio for vortioxetine was not significantly different between these treatment groups [*t*(14) = 0.12, n.s.].

**Table 2:** No pharmacokinetic interactions are present between acute vortioxetine and MK-801.

Treatment Group	Vortioxetine			MK-801		
	Plasma (μM)	Brain (μmol/kg)	Brain:Plasma	Plasma (μM)	Brain (μmol/kg)	Brain:Plasma
Vortioxetine (10 mg/kg 60 min s.c.)	2.3 ± 0.1	44.6 ± 7.6	20.1 ± 3.6	0	0	0
MK-801 (0.1 mg/kg, 30 min s.c.)	0	0	0	67 ± 10	960 ± 220	15.2 ± 2.6
Vortioxetine (10 mg/kg, 60 min s.c.) + MK-801 (0.1 mg/kg, 30 min s.c.)	2.3 ± 0.15	43.4 ± 3.5	19.6 ± 1.5	66 ± 8.6	1340 ± 240	19.7 ± 2.3

*Using administration routes, timing, and doses identical to those used in the visual signal detection task, vortioxetine administration had no significant effects on MK-801 plasma exposure, brain exposure, or brain:plasma ratio. Similarly, MK-801 had no significant effects on vortioxetine plasma exposure, brain exposure, or brain:plasma ratio. Data are presented as Mean ± SEM. The sample size was 7–8 animals per group.*

#### MK-801 Exposure

Similarly, MK-801 exposure data in animals treated with vehicle + 0.1 mg/kg MK-801 was not significantly different from that observed in animals treated with 10 mg/kg vortioxetine + 0.1 mg/kg MK-801 in plasma [*t*(14) = 0.02, n.s.], or brain [*t*(13) = 1.09, n.s.]. Furthermore, the brain:plasma ratio for MK-801 was not significantly different between these two treatment groups [*t*(13) = 1.30, n.s.].

## Discussion

This study examined the effects of vortioxetine and MK-801, administered alone and in combination, on attention (measured in the VSDT) and locomotor activity. We observed that the selective glutamatergic NMDA receptor antagonist MK-801 impaired VSDT performance and elicited marked hyperlocomotion at the doses used. Vortioxetine had no effect on VSDT performance alone. However, when administered in combination with MK-801, vortioxetine pre-treatment exacerbated MK-801’s effects. This effect cannot be explained by a pharmacokinetic interaction, given that vortioxetine pretreatment had no significant effects on MK-801 exposure in brain or plasma. Thus, the interaction between vortioxetine and MK-801 must be pharmacodynamic in nature. We further found that vortioxetine does not affect MK-801-induced hyperlocomotion, which may imply that vortioxetine’s interaction with MK-801 is not system-wide, but instead is circumscribed to specific brain circuits.

### Vortioxetine Does Not Alter Attention Performance Under Normal Conditions

We observed that 10 mg/kg vortioxetine (1 h s.c.), which represents the top of the clinically relevant dose range based on SERT occupancy (Stenkrona et al., 2013; Leiser et al., 2014), does not alter VSDT performance in normal rats. This result is consistent with previous work from this laboratory, which also demonstrated that this vortioxetine dose does not alter VSDT performance (Pehrson et al., 2016a). Laursen et al. (2017) investigated vortioxetine’s effects in a two-tone auditory discrimination task, another putative measure of attention performance, and found that vortioxetine did not alter rodents’ performance at the 3 and 10 mg/kg doses. It should be noted that vortioxetine significantly increased the amplitude of hippocampal P3 ERPs, which may be related to attention processing. However, these data suggest that vortioxetine consistently does not affect behavioral attention measures in unimpaired animals.

This conclusion should be viewed cautiously from several perspectives. First, to date vortioxetine has been assessed in a limited number of validated behavioral attention models. It remains possible, although unlikely, that vortioxetine will alter attention in other models. Additionally, vortioxetine has yet to be assessed in a model of MDD-related attention deficits. Finally, the current study evaluated vortioxetine’s effects under acute dosing conditions rather than chronic administration, which would be more consistent with its clinical use. Thus, in order to fully understand whether vortioxetine has effects on attention function that could be relevant for MDD, future studies should focus on evaluating its effects in MDD-related models of attention deficit using chronic administration.

There is a paucity of studies that have investigated antidepressant effects on attention, and the available data has generally used normal animals. Several research groups have demonstrated that serotonin reuptake inhibitors such as citalopram (Baarendse and Vanderschuren, 2012; Humpston et al., 2013), fluoxetine (Humpston et al., 2013), or paroxetine (Humpston et al., 2013) either do not modify attention performance, or induce impairments such as increased omissions or response latency. From this perspective, vortioxetine’s limited effects on attention in normal rodents are consistent with the actions of other serotonergic antidepressants.

### Acute Treatment With MK-801 Impairs VSDT Performance and Induces Hyperlocomotion

We observed that acute administration of 0.1 mg/kg MK-801 induced a signal intensity-dependent impairment in hits, increased the response latency for hits, and reduced correct rejections. These effects are consistent with the literature of non-competitive NMDA receptor antagonist effects on attention function. Other labs have demonstrated that acute MK-801 (Howe and Burk, 2007; Rezvani et al., 2009; Levin et al., 2014) or ketamine treatment (Hillhouse et al., 2015) induces similar VSDT impairments. Furthermore, several labs have demonstrated similar MK-801-induced impairments in alternate behavioral attention models after either systemic (Auger et al., 2017) or intra-cerebral microinjection into the mPFC (Auger et al., 2017), or the anterior cingulate cortex (Pehrson et al., 2013a).

In the locomotor activity portion of the current study, we demonstrated that systemic treatment with MK-801 induced hyperlocomotion in a manner that is consistent with published literature (Amalric et al., 1994; Ogren and Goldstein, 1994; Hatip-Al-khatib et al., 2001; Scorza et al., 2008).

### Acute Pretreatment With Vortioxetine Selectively Potentiates MK-801’s Effects on Attention via a Pharmacodynamic Interaction

Contrary to our hypothesis that vortioxetine administration would attenuate MK-801’s effects on VSDT performance, we observed that vortioxetine potentiated these effects on several VSDT dependent measures, including hits, correct rejections, response latencies, and omissions. However, when the combination of vortioxetine and MK-801 was administered in locomotor activity, we observed no difference compared to the MK-801 alone treatment group. We consider it unlikely that this lack of a difference in hyperlocomotion is due to a ceiling effect, given that other research groups have observed MK-801-induced increases in horizontal activity up to 0.5 mg/kg (Amalric et al., 1994). Thus, vortioxetine appears to selectively interact with MK-801, potentiating its effects on attention but not locomotion.

This result is somewhat surprising in light of evidence that acute or subchronic vortioxetine treatment reverses subchronic phencyclidine-induced impairments in memory and the AST (Pehrson et al., 2018). Although the AST and VSDT examine separate cognitive domains, executive function and attention, respectively, portions of both the AST and the VSDT are thought to depend on frontal cortex function. Thus, based on these data it is apparent that vortioxetine administration does not universally reverse the effects of NMDA receptor antagonists on frontal cortex-dependent cognitive tasks. Instead, it appears that vortioxetine has complex interactions with non-competitive NMDA receptor antagonists. Based on the accumulated data, the mechanisms driving these differences in vortioxetine’s effects are unclear. It may be that vortioxetine modulates the specific frontal cortex sub-regions that these separate cognitive domains depend on differently, or it may be a difference in vortioxetine’s effects under the acute vs. subchronic effects of non-competitive NMDA receptor antagonists. More study is required to differentiate between these possibilities.

We considered two possible mechanistic explanations for the selective vortioxetine-MK-801 interaction mentioned above. First, vortioxetine and MK-801 might interact pharmacokinetically, wherein the presence of vortioxetine would increase brain MK-801 exposure. Alternately, the vortioxetine-MK-801 interaction could be pharmacodynamic in nature, wherein vortioxetine’s receptor actions would increase MK-801’s ability to access its binding site. In light of the selective effect of vortioxetine on MK-801-induced attention impairments but not hyperlocomotion, we considered it unlikely that a pharmacokinetic interaction was the culprit. If vortioxetine caused an increase in MK-801 brain exposure, it would occur everywhere in the brain, and therefore would not be selective for one behavior over another.

In order to empirically evaluate whether a pharmacokinetic interaction between vortioxetine and MK-801 was present, we examined plasma and brain exposure after administration of vortioxetine, MK-801, or combinations thereof. We found no differences in MK-801 or vortioxetine exposure between these conditions. Therefore, the vortioxetine-MK-801 interaction observed in VSDT impairments must be pharmacodynamic in nature.

### A Proposed Pharmacodynamic Mechanism for Vortioxetine’s Selective Influence on MK-801-Induced Attention Impairments: Regionally Selective Increases in Depolarization of NMDA Receptor-Expressing Neurons

We propose that vortioxetine’s pharmacodynamic interaction with MK-801 is mediated by a regionally selective increase in membrane depolarization in NMDA receptor-expressing neurons. This theoretical mechanism is based on three premises: (1) MK-801-induced behavioral alterations in attention and locomotor behavior depend on separate brain circuits, (2) MK-801’s pharmacological effects are receptor activation- and voltage-dependent, (3) vortioxetine may induce regionally selective increases in excitatory neurotransmission, thereby having different effects on neuronal activity in different brain regions. Evidence in favor of each of these premises is presented below.

Premise 1: MK-801-induced behavioral alterations in attention and locomotion depend on separate brain circuits.

It is well known that systemic MK-801 administration impairs attention models and induces hyperlocomotion. MK-801’s brain region-specific effects in these behavioral models are less well characterized, however some data is available. In behavioral attention measures such as the 3 choice serial reaction time (3CSRT) task, electrophysiological data demonstrates that the anterior cingulate cortex (ACC) and medial prefrontal cortex (mPFC) play a role in representing attention-relevant information in rats (Totah et al., 2009), and intra-ACC MK-801 injections selectively increased omissions in the 3CSRT task without altering the accuracy (Pehrson et al., 2013a). In the sustained attention task, intra-mPFC MK-801 injections impaired vigilance during high-difficulty conditions (Auger et al., 2017). We could not find any study that evaluated non-competitive NMDA receptor antagonists in the VSDT using region-specific microinjections; thus, it is unknown whether these effects translate to this attention model. But overall, NMDA receptors in frontal cortex regions seem to mediate portions of attention performance.

However, the available data suggest that the frontal cortex does not mediate MK-801-induced hyperlocomotion, whereas the nucleus accumbens (NAc) may play an important role. Scorza et al. (2008) demonstrated that systemic MK-801 injections induced robust locomotor increases, and that electrolytic mPFC lesions did not alter MK-801-induced hyperlocomotion. However, MK-801 infusions into the NAc increased locomotor behavior (Raffa et al., 1989), and 6-OHDA lesions of the NAc block systemic MK-801-induced hyperlocomotion (Hatip-Al-khatib et al., 2001). Taken together, these data suggest that although MK-801-induced attention impairment are mediated in part by the frontal cortex, its effects on locomotion are at least partially mediated by subcortical regions such as the NAc.

Premise 2: MK-801 has receptor activation- and voltage-dependent pharmacological effects.

Early studies investigating the mechanistic effects of non-competitive NMDA receptor antagonists such as MK-801 suggest that these drugs have receptor activation- and voltage-dependent pharmacological effects. Molecular pharmacology studies demonstrate that the binding of [^3^H]MK-801 is facilitated by NMDA receptor orthosteric agonists, and by the co-agonist glycine (Foster and Wong, 1987). Furthermore, competitive antagonists such as 2-amino-5-phosphonovalerate reduce MK-801 binding (Foster and Wong, 1987). Electrophysiological studies have also demonstrated that MK-801’s ability to block the NDMA receptor channel is both voltage- and NMDA receptor agonist-dependent (Huettner and Bean, 1988; Halliwell et al., 1989). These voltage-dependent pharmacological actions of MK-801 are due to the position of the non-competitive NMDA receptor antagonist binding site being positioned behind a Mg^2+^ ion inside the channel (Huettner and Bean, 1988). Thus, MK-801 binding requires receptor activation, and sufficient cellular membrane depolarization to expel the Mg^2+^ ion from the channel.

Premise 3: Vortioxetine induces indirect increases in excitatory neurotransmission that are likely to be regionally selective.

Although vortioxetine has a purely serotonergic pharmacological profile within the clinically relevant dose range (Leiser et al., 2014), there is an accreting narrative suggesting that this compound exerts complex effects on excitatory and inhibitory neurotransmission that may vary markedly across brain regions. At the doses used here, vortioxetine fully antagonizes 5-HT_3_ receptors (Leiser et al., 2014), which are excitatory ion channels selectively expressed in non-parvalbumin-positive GABAergic interneurons (Morales et al., 1996; Morales and Bloom, 1997). Immunohistochemical and autoradiographic expression studies have demonstrated that 5-HT_3_ receptors have a circumscribed expression pattern, with relatively high expression in the mPFC, but low expression levels in striatal regions such as the NAc (Morales et al., 1998). Thus, 5-HT_3_ receptors can be thought of as a serotonin-mediated fast excitatory drive on non-fast-spiking GABAergic interneurons. In the frontal cortex and hippocampus, where these GABAergic interneurons modulate glutamatergic pyramidal neuron function, 5-HT_3_ receptor antagonism can be expected to disinhibit glutamate neurotransmission (reviewed in Dale et al., 2016; Pehrson et al., 2016b). However, in the NAc, where there is very little 5-HT_3_ receptor expression, a local disinhibition of this sort would not be expected to occur (Pehrson et al., 2016b).

Recent mechanistic data from electrophysiology studies support portions of this theory. Dale et al. (2014) demonstrated in hippocampal slices that vortioxetine blocks 5-HT_3_ receptor agonist-evoked inhibitory postsynaptic currents (IPSCs) recorded in pyramidal neurons, suggesting that vortioxetine blocked 5-HT_3_ receptor-mediated GABAergic neurotransmission (also see (Dale et al., 2017). Additionally, a separate group of researchers found that acute or chronic vortioxetine increased cortical pyramidal neuron firing rates (Riga et al., 2016, 2017). This effect was also observed using the selective 5-HT_3_ receptor antagonist ondansetron, but not the serotonin reuptake inhibitor escitalopram (Riga et al., 2016). Finally, Chakroborty et al. (2017) demonstrate that vortioxetine suppresses medium spiny neuron activation in the NAc, probably by exciting local fast-spiking interneurons. Thus, vortioxetine’s effects on excitatory and inhibitory neurotransmission appear to be regionally dependent.

If these premises are correct, then a plausible hypothesis for vortioxetine’s selective potentiation of MK-801-induced behavioral effects can be derived. Given that MK-801 has activity-dependent pharmacological effects at the NMDA receptor, and that vortioxetine indirectly increases mPFC glutamate neurotransmission, vortioxetine may potentiate MK-801 induced attention impairments by increasing depolarization of NMDA receptor-expressing cells, increasing access to the MK-801 binding site. This mechanism would likely be active in the mPFC (Riga et al., 2016, 2017), a brain region that mediates at least some MK-801 effects on attention performance (Totah et al., 2009; Pehrson et al., 2013a; Auger et al., 2017) but is irrelevant for MK-801’s locomotor effects. Furthermore, vortioxetine’s 5-HT_3_ receptor-dependent disinhibitory effects would be inactive in the NAc, which may mediate a portion of MK-801-induced hyperlocomotion (Raffa et al., 1989; Hatip-Al-khatib et al., 2001). Thus, it is possible that the observed selective interaction between vortioxetine and MK-801 is caused by regionally circumscribed increases of excitatory neurotransmission by vortioxetine. However, it must be stressed that these ideas are merely a theory, and more empirical research must be performed to evaluate whether this theoretical mechanism is correct.

### Limitations and Future Research

The current study is limited from several perspectives. First, as an evaluation of vortioxetine’s effects on attention, this study has some features that limit its translational value. Vortioxetine’s affinity at 5-HT_1A_ and 5-HT_7_ receptors is markedly lower in rodents than in humans (Sanchez et al., 2015). Thus, if 5-HT_1A_ receptor agonism or 5-HT_7_ receptor antagonism modulate mechanisms related to attention or non-competitive NMDA receptor antagonists, then the effects observed here may not translate to humans. Second, the limited number of MK-801 and vortioxetine doses used in the current study do not allow for a complete understanding of the pharmacodynamic mechanisms controlling the observed interactions between these compounds. Furthermore, vortioxetine is used under chronic administration conditions in clinical situations, but was acutely administered in the current study. Therefore, it is not clear to what extent vortioxetine’s acute effects on attention performance are relevant to clinical situations. Finally, the proposed mechanistic theory regarding the pharmacodynamic interaction between vortioxetine and MK-801 has not been properly evaluated in this study, and thus it must be viewed with caution until more studies can be conducted.

In light of these limitations, several suggestions for future research can be made. First, future studies should replicate the observations that vortioxetine does not modulate attention function, and that vortioxetine selectively potentiates MK-801’s effects, in other attention models. Future studies should also examine whether these results can be replicated under multiple dose combinations of vortioxetine and MK-801, and under a regimen of chronic vortioxetine administration. Finally, future work should evaluate which of vortioxetine’s receptor mechanisms are involved in the pharmacodynamic interaction with MK-801.

## Conclusion

The current study has several notable findings. First, we observed that acute vortioxetine treatment in unimpaired adult rodents does not modulate performance in an attention model. We further observed that vortioxetine selectively potentiates MK-801-induced impairments in VSDT performance without altering its effects on locomotion, and found that this interaction was not due to altered MK-801 exposure. Given the activity-dependent pharmacological actions of non-competitive NMDA receptor antagonists and observations that vortioxetine increases cortical pyramidal neuron firing, it may be that the vortioxetine-MK-801 interaction is due to a regionally selective increase in excitatory neurotransmission, however, more research is required to test this theory.

## Author Contributions

All the authors provided significant contributions to the submitted manuscript. TH, CS, JP, and AP designed the experiments. TH, CM, DS, MC, and AP conducted all of the research experiments. TH and AP wrote the first draft of the manuscript. All authors contributed to the final draft of the manuscript.

## Conflict of Interest Statement

MC, AP, and CS were employees of Lundbeck Research USA, Inc. during data collection. JP received monetary support from Lundbeck Research USA, Inc. to conduct this research. The remaining authors declare that the research was conducted in the absence of any commercial or financial relationships that could be construed as a potential conflict of interest.
